# The Effect of Fascia Iliaca Block on Post-Operative Pain in Elderly Patients (≥70 Years Old) Undergoing Hip Fracture Surgery: A Systematic Review

**DOI:** 10.7759/cureus.108450

**Published:** 2026-05-07

**Authors:** Edward S.Y. Yong, Chern Yea Pang

**Affiliations:** 1 Trauma and Orthopaedics, Newcastle upon Tyne Hospitals NHS Foundation Trust, Newcastle, GBR; 2 Anaesthesiology, South Tyneside and Sunderland NHS Foundation Trust, Sunderland, GBR

**Keywords:** acute pain, elderly patients, fascia iliaca compartment block (ficb), hip and proximal femur trauma, numeric rating scale (nrs), orthogeriatric care, orthopaedics & traumatology, regional anesthesiology, surgical hip repair, visual analogue scale (vas)

## Abstract

The aim of this systematic review was to compare post-hip surgery pain outcomes in the elderly population after a fascia iliaca compartment block (FICB) against conventional systemic analgesia (CSA). Our primary outcome was post-operative static and dynamic visual analogue scales (VAS)/numerical rating scales (NRS) as our validated pain measurements. Secondary outcomes were post-operative opioid consumption, incidence of post-operative delirium, and average length of hospital stay. A systematic search strategy was conducted in PubMed, EMBASE, and the Cochrane Central Register of Controlled Trials (CENTRAL) from database inception to 6th April 2026. We accepted observational studies and randomised controlled trials comparing the post-operative pain outcomes in elderly patients who received an FICB for their hip fracture against those receiving CSA without any nerve blocks. Risk of bias was assessed with the Cochrane Risk-of-Bias 2 tool for randomised trials (RoB2) and the Newcastle-Ottawa Scale (NOS), depending on the type of study. Certainty assessments were conducted using the Grading of Recommendations Assessment, Development, and Evaluation (GRADE) tool. A narrative synthesis was performed without a meta-analysis due to the clinical and methodological heterogeneity seen amongst outcome parameters and measured effects. A total of 228 studies were identified, of which there were 143 from CENTRAL, 45 from EMBASE, and 40 from PubMed. Eleven studies were included after the literature search. The 11 eligible studies included seven randomised controlled trials (63.6%) and four non-randomised studies of interventions (36.4%), totalling 1,844 participants, of which 1,068 (58%) were from control/CSA groups and 776 (42%) were from intervention/FICB groups, wherein the mean age of all participants was 78.7 years. Pain outcome analysis favoured the use of the FICB. The average static pain intensity difference (Δ static pain) in the control group (CG) between POD1 and pre-operative levels was as follows: CG Δ static pain of -2.55 per VAS and -2.67 per NRS. For the FICB group, it was as follows: FICB Δ static pain of -2.86 per VAS and -3.36 per NRS. The average dynamic pain intensity difference (Δ dynamic pain) in the CG between POD1 and pre-operative levels was as follows: CG Δ dynamic pain of -2.83 per VAS. For the FICB group, it was as follows: FICB Δ dynamic pain of -3.82 per VAS. There were no NRS data for the dynamic pain group. Secondary outcome findings were varied in evidence certainty, although a notable point was the significant opioid-sparing effect seen in FICB patients, whilst evidence pertaining to the other outcomes remained equivocal. The FICB, as part of a multimodal analgesia regimen, offers meaningful clinical benefits over CSA in elderly hip fracture patients requiring surgery. With moderate to high evidence certainty, FICB reduced both static and dynamic pain scores and consequently reduced post-operative opioid consumption as well. Average LOS had moderate evidence certainty, suggesting it is unaffected by the inclusion of FICB when compared to the CSA group. Other secondary outcomes, such as post-operative delirium, nausea, vomiting, and pruritus, were deemed inconclusive due to insufficient evidence or low to very low evidence certainty.

## Introduction and background

Elderly patients (aged ≥70 years) are increasingly sustaining neck of femur/hip fractures, with age-standardised prevalence rates climbing from 3,521.6 to 4,366.8 per 100,000 and age-standardised incidence rates increasing from 1,866.9 to 2,218.5 per 100,000 when comparing data from 1990 to 2021 [1]. This could be due to susceptibility factors such as, but not limited to, increasing age, poor bone nutrition due to malnutrition, iatrogenic or physiologically degraded gait and balance ability leading to an increased propensity for falls, and, specifically for post-menopausal females, menopausal biochemical changes leading to accelerated osteoporosis [2]. These factors are involved in both an increased risk of falls and an increased fracture risk during such falls. Hip fractures also significantly affect the quality of life of patients due to pain and mobility degradation and require surgical interventions more often than not. One study found that post-hip fracture, long-term functional outcomes in older patients were significantly deteriorated from baseline in multiple aspects (such as care home admissions, walking aid requirements, and general physical quality of life), and the mortality rates after 30-day, 1-year, and 5-year follow-ups were 7.9%, 37.0%, and 69.4%, respectively, underscoring the importance of optimised peri-operative treatment protocols in this patient group for this injury [3]. Secondary to that, the pain surrounding a hip fracture is especially significant, given the size and anatomy of the femur, its complex sensory innervation, and its weight-bearing nature. In addition, the elderly are also particularly susceptible to the adverse effects of systemic opioids, which could include delirium, respiratory depression, and constipation, due to physiological age-related changes in pharmacokinetics such as an increased volume of distribution associated with increased adipose tissue, reduced glomerular filtration rates, and reduced hepatic blood flow [4]. As a result, a variety of regional block techniques are commonly used as part of multimodal analgesia strategies in certain centres to increase comfort and minimise overall opioid consumption by these hip fracture patients, who are usually elderly. One example would be the fascia iliaca compartment block (FICB), which targets the integrated innervation of the hip. Successful FICBs could anesthetise the femoral, lateral femoral cutaneous, genitofemoral, and sometimes the obturator nerve, making it an effective block choice for most lower limb orthopaedic procedures [5]. Other examples include the peri-capsular nerve group block (PENG), lateral femoral cutaneous nerve block (LFCN), and the femoral nerve block (FNB) [6]. Although there is an ongoing general consensus supporting the use of the regional nerve blocks [7], we would like to specifically investigate the efficacy of the FICB with regards to post-operative pain outcomes, amongst others, in the elderly population who have sustained hip fractures, when compared against the current conventional systemic analgesia (CSA).

As a whole, this review aims to compile and provide further evidence and clarity surrounding the efficacy of the FICB across multiple outcomes to continue to guide the advent of regional nerve blocks in hip fracture pathways worldwide.

## Review

Methodology

The reporting in this systematic review followed the 27-item Preferred Reporting Items for Systematic Reviews and Meta-Analyses (PRISMA 2020) checklist and its associated PRISMA-Abstract extension [8]. The research question was also compliant with the Population, Intervention, Comparison, and Outcome (PICO) framework. The research protocol that this study adhered to was developed and published beforehand in the International Prospective Register of Systematic Reviews (PROSPERO, CRD420261361789). Any disagreements or doubts raised at any stage of this review were discussed in person and resolved between the authors (E.Y.S.Y. and P.C.Y.), with an option to involve a third member for adjudication if needed.

Search Strategy

We performed a systematic search of MEDLINE (via PubMed), EMBASE (via Ovid), and the Cochrane Central Register of Controlled Trials (CENTRAL), from database inception to 6th April 2026. We also performed backward citation mining in all included papers at the full-text screening stage. The search strategy that we composed was made without the assistance of a librarian, although we best endeavoured to be as comprehensive as possible. We employed the use of controlled vocabulary such as Boolean Operators, Medical Subject Headings (MeSH) for use in PubMed and CENTRAL, EMTREE terms in EMBASE, truncations for improved sensitivity throughout, and field tags in PubMed and EMBASE. Notably, multiple papers were identified from CENTRAL, which originated from the Chinese Clinical Trial Registry (ChiCTR) or the Thai Clinical Trial Registry (TCTR), all of which we attempted to source English versions for screening, and we did not outright exclude trials that came from international trial registries unless an English language version of the full text could not be retrieved. The complete search strategy we used is given in Table 1.

**Table 1:** Search strategy utilised

Database	Search Strategy
PubMed	("Fascia Iliaca Block"[Mesh] OR "fascia iliaca block"[tiab] OR "fascia iliaca compartment block"[tiab] OR FICB[tiab] OR FIB[tiab]) AND ("Hip Fractures"[Mesh] OR "hip fracture*"[tiab] OR "femoral neck fracture*"[tiab] OR "intertrochanteric fracture*"[tiab]) AND ("Aged"[Mesh] OR elderly[tiab] OR geriatric*[tiab] OR aged[tiab] OR "over 70"[tiab]) AND ("Postoperative Pain"[Mesh] OR "postoperative pain"[tiab] OR "post-operative pain"[tiab] OR "pain score*"[tiab] OR VAS[tiab] OR NRS[tiab]) Filters applied: Clinical Trial, Observational Study, Randomized Controlled Trial.
EMBASE (Ovid)	('fascia iliaca block'/exp OR 'fascia iliaca block':ti,ab OR 'fascia iliaca compartment block':ti,ab OR FICB:ti,ab OR FIB:ti,ab) AND ('hip fracture'/exp OR 'hip fracture*':ti,ab OR 'femoral neck fracture*':ti,ab OR 'intertrochanteric fracture*':ti,ab) AND ('aged'/exp OR elderly:ti,ab OR geriatric*:ti,ab OR aged:ti,ab OR 'over 70':ti,ab) AND ('postoperative pain'/exp OR 'postoperative pain':ti,ab OR 'post-operative pain':ti,ab OR 'pain score*':ti,ab OR VAS:ti,ab OR NRS:ti,ab)
CENTRAL (Cochrane Library)	("fascia iliaca block" OR "fascia iliaca compartment block" OR FICB OR FIB) AND ("hip fracture" OR "femoral neck fracture" OR "intertrochanteric fracture") AND (elderly OR geriatric OR aged OR "over 70") AND ("postoperative pain" OR "post-operative pain" OR "pain score" OR VAS OR NRS)

Eligibility Criteria

Our review included any relevant peer-reviewed papers found during our systematic search with regards to determining if the FICB improves post-operative pain outcomes compared to standard or alternative systemic analgesic techniques in elderly patients (≥70 years old) undergoing surgery for hip fractures.

Hence, our inclusion criteria were as follows:

Population: hip fracture patients aged ≥70 undergoing surgery (or studies with mean/median age ≥70, or extractable ≥70 age subgroup data)

Intervention: use of the FICB specifically (any technique/type)

Comparator: standard analgesia (placebo/sham/no block/other systemic anaesthesia)

Outcome: post-operative pain with visual analogue scales (VAS)/numerical rating scales (NRS) (at rest and/or on movement)

English language papers not requiring translation and study types such as randomised controlled trials (RCTs) and non-randomised studies of interventions (NRSIs), examples being retrospective, prospective case-control, and cohort studies.

Our exclusion criteria were as follows: patients with non-hip fractures or for non-operative management, elective surgery cases, studies where FICB was compared to other regional block techniques without extractable CSA control groups, studies with no pain outcomes, studies where we were unable to isolate the effect of the FICB, case reports/series (<10 patients), review articles, editorials, letters, and conference abstracts without full data.

We also accepted studies which reported on certain pre-defined secondary outcomes, such as post-operative delirium incidence, average length of hospital stay (LOS), and post-operative opioid consumption.

Study Selection/Screening

The resulting studies were then imported into a reference manager, EndNote (EndNote Online Classic, Clarivate Analytics, UK), where they were compiled and automatically deduplicated prior to being exported to Rayyan AI (Rayyan Systems Inc., Qatar) as our primary screening and organisation software.

**Figure 1:**
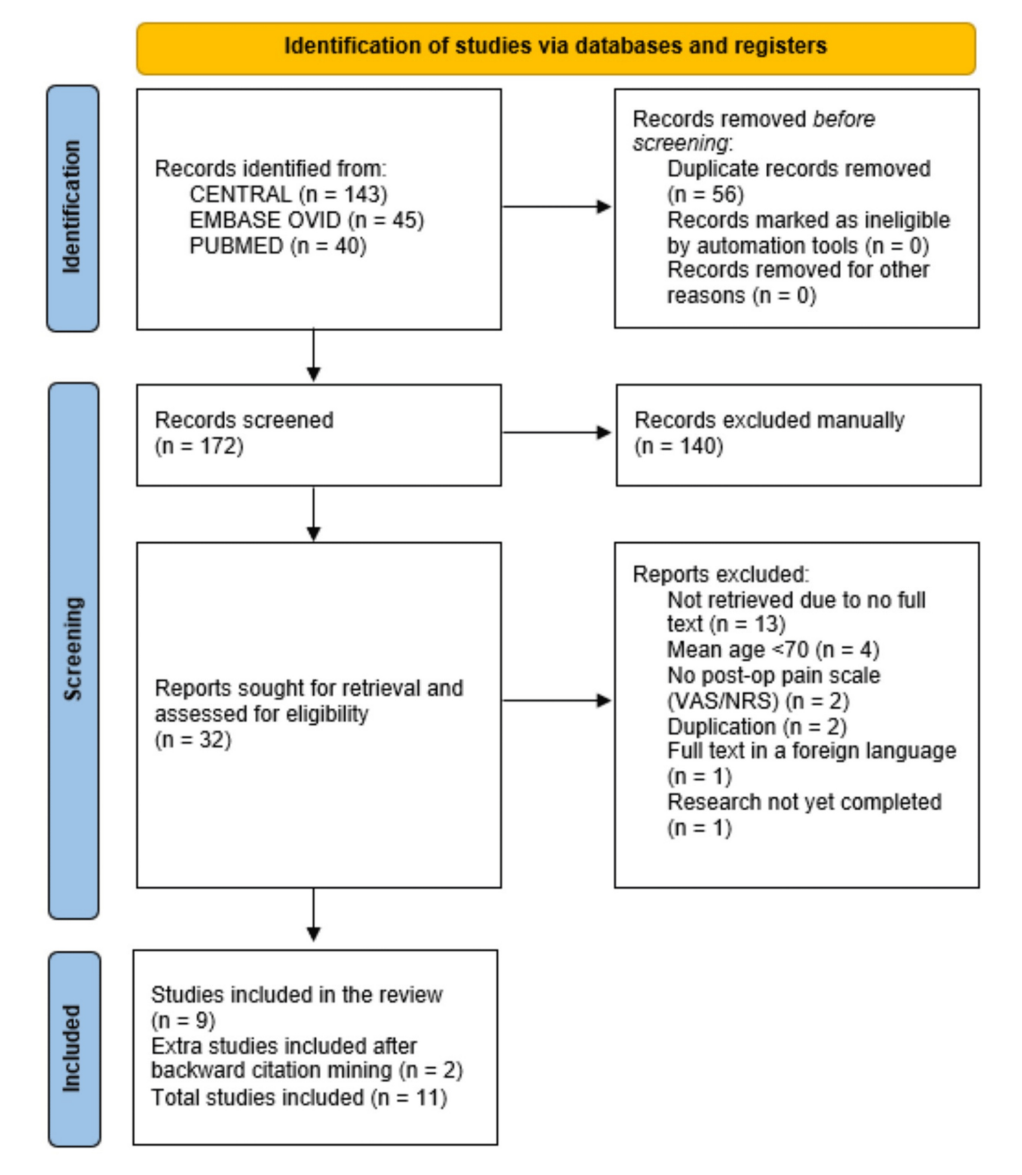
PRISMA flow chart PRISMA, Preferred Reporting Items for Systematic Reviews and Meta-Analyses

The authors (E.Y.S.Y. and P.C.Y.) first piloted the screening process to resolve consensus/interpretation discrepancies and then individually and systematically screened through the deduplicated studies at a title/abstract level initially, with disputes later resolved through in-person discussions, and again at a full-text level using the same piloting and dispute resolution discussion method. Strict adherence to the aforementioned selection criteria was upheld throughout all stages of screening. A third-party adjudicator was available but not needed. The detailed breakdown of this process, including reasons for full-text rejections, can be found in the PRISMA flow chart in Figure 1. 

Data Extraction and Management

The authors (E.Y.S.Y. and P.C.Y.) then performed structured data extraction on the final set of included studies imported from Rayyan AI using Covidence (Covidence systematic review software, Veritas Health Innovation, Melbourne, Australia). Both authors discussed and formulated a data extraction strategy within Covidence, which included key study characteristics and outcome measurements beforehand. Data extraction was first piloted to resolve unforeseen consensus and interpretation discrepancies and then subsequently performed using the ‘independent dual-extraction plus adjudication’ method. At completion, a final consensus was sought via an in-person discussion to resolve discrepancies.

Extracted key data elements in Covidence included study details (year, author, study type, and country of study), population details (their inclusion and exclusion criteria, total sample size, and sex demographics), baseline characteristics of the control and FICB groups (mean ages, American Society of Anaesthesiologists [ASA] status, surgical procedure type breakdown, type of anaesthesia used, and sample size), the complete analgesia regimen used for both the control and FICB groups, outcome types, their respective values, specific VAS/NRS values at all available post-operative time points, and summaries of their conclusions and study limitations. All extracted data were subsequently tabulated in Microsoft Excel (Microsoft Corp., Redmond, WA). The results were then reviewed and discussed by both authors (E.Y.S.Y. and P.C.Y.).

Risk of Bias and Certainty Assessment

The reviewers (E.Y.S.Y. and P.C.Y.) then individually performed a comprehensive and validated risk-of-bias assessment on the included studies using the Cochrane Risk-of-Bias 2 (RoB2) tool for included RCTs and the Newcastle-Ottawa Scale (NOS) for included NRSIs [9,10]. For the purposes of the RoB2 assessment, we adopted suggestions by Cochrane to optimise the use of the tool and explicitly chose the ‘effect of assignment to intervention/intention to treat effect’ version of domain 2, as we were assessing the effects of an intervention [11]. The RoB2 tool was also specifically applied by the reviewers to the primary outcome, that is, the post-operative VAS/NRS scores, and not with regards to the study as a whole, as also advised by Cochrane [11]. The risk-of-bias assessment was piloted by the reviewers to resolve consensus/interpretation-related issues before the RCTs were judged according to the official RoB2 guidance crib sheet to ensure standardised decision-making throughout [12]. Piloting and validated external guidance were likewise used for NOS when assessing the NRSIs to improve standardisation [13].

The reviewers (E.Y.S.Y. and P.C.Y.) then discussed and formulated the five most review-relevant outcomes for a ‘certainty of evidence’ assessment to be carried out on, and these were as follows: day 1 post-operative pain scores at rest, day 1 post-operative pain scores on movement, post-operative incidence of delirium, day 1 post-operative opioid consumption, and average LOS. The Grading of Recommendations Assessment, Development, and Evaluation (GRADE) tool was first piloted by the reviewers to resolve unforeseen interpretation/consensus issues and was then used to individually perform a formal certainty assessment on these outcomes. GRADE guidance by the Cochrane Consumers and Communication Group (CCCG) was utilised to best ensure standardised decision-making [14]. The final certainty outcomes were then tabulated as recommended by GRADE policies [15].

Data Synthesis and Analysis

The reviewers (E.Y.S.Y. and P.C.Y.) then performed a qualitative/narrative analysis on this systematic review. The reasons for not performing a meta-analysis were due to the clinical and methodological heterogeneity seen amongst outcome parameters and measured interventions across included papers, which would be further detailed in the Results section. Notably, we also chose not to directly interpret VAS and NRS as interchangeable outcomes, which led to increased heterogeneity, as a 2024 study had shown poor agreement in pain measurements between these scales in older adults [16]. Narrative analysis was done through data extraction as described above, where the two authors (E.Y.S.Y. and P.C.Y.) subsequently analysed and discussed. Conclusions were then drawn from the results and discussion as described in the relevant sections below.

Results

A total of 228 studies were identified from the search strategy, of which 143 were from CENTRAL, 45 were from EMBASE (Ovid), and 40 were from PubMed (Medline). The automated deduplication software in EndNote removed 50 duplicate studies, whilst the human-checked deduplication software in Rayyan removed six more duplicates, leaving 172 studies for title/abstract level screening as described in the Methods section above. After discussion and consensus, title/abstract screening removed 140 studies, leaving 32 studies for full-text retrieval and eligibility assessment as described in the Methods section above. After full-text retrieval, assessment, discussion, and consensus, 23 studies were excluded, leaving nine studies for eventual inclusion. Notable exclusions involved several studies that were accepted for full-text retrieval but were subsequently excluded due to a mean age of <70 years [17-20] or due to a lack of pain score recorded [21]. We then went a step further to perform backwards citation mining on all possible papers from the 32 studies that were included at the full-text stage, which yielded two further studies that were eligible to be included after full-text screening. A final total of 11 studies were included after all steps of the systematic literature search were completed [22-32]. This search is illustrated in the PRISMA flow chart in Figure 1.

The 11 eligible studies included seven RCTs (63.6%) [22,23,27,29-32] and four NRSIs (36.4%) [24-26,28], totalling 1,844 participants, of which 1,068 (58%) were from the control/CSA groups and 776 (42%) were from the intervention/FICB groups, wherein the mean age of all participants was 78.7 years. Six (54.5%) of the studies were conducted in China [22,23,25,27,29,31], two (18.2%) in Turkey [28,30], two (18.2%) in the United States [24,26], and one (9.1%) in Korea [32]. Five (45.5%) studies utilised spinal anaesthesia for the surgery [23,27,30-32], four (36.4%) studies utilised general anaesthesia [22,25,28,29], one (9.1%) used both general anaesthesia and spinal anaesthesia [26], and one (9.1%) study did not disclose their anaesthesia modality [24]. Five (45.5%) studies performed their FICB pre-operatively, three (27.3%) studies performed their FICB post-operatively [22,30,32], two studies (18.2%) performed their FICB intra-operatively [27,28], and one (9.1%) study did not standardise their FICB administration timing [26]. Almost all (n = 9, 81.8%) studies utilised ultrasound-guided FICB insertion techniques, one (9.1%) study utilised the ‘double-pop’ landmark-guided FICB insertion technique [27], and one (9.1%) study did not disclose their insertion technique [22]. For pain reporting, most (n = 8, 72.7%) studies utilised the VAS scale, whilst the remainder (n = 3, 27.3%) studies used the NRS scale [22,26,30]. Only one (9.1%) study utilised a sham/placebo block for true blinding [23], whilst all (n = 10, 90.9%) other studies did not. All (n = 11, 100%) studies reported overwhelmingly positive final conclusions supporting the use of FICB as part of multimodal analgesia for elderly hip fractures. These results can be derived from the summary table of key study characteristics presented in Table 2.

**Table 2:** Key study characteristics of the included studies BD, twice daily; FICB, fascia iliaca compartment block; GA, general anaesthesia; IM, intramuscular; IV, intravenous; NaCl, sodium chloride; NRS, numerical rating scale; PCA, patient-controlled analgesia; PO, per oral; POD1, post-operative day 1; PRNQDS, as per needed four times a day; RA, rescue analgesia; RCT, randomised controlled trials; US, ultrasound; VAS, visual analogue scale

Author (year)	Type of study	Number of patients (control/FICB)	Mean age (years old)	Anaesthesia type	Pain management (control)	Timing (FICB)	Technique (FICB)	Dosage (FICB)	Pain assessment tool	Comparison	Conclusion
Nie et al. (2015) [22]	RCT	n = 104 (53/51)	70.65	GA	IV PCA (fentanyl + tropisetron) + RA (acetaminophen, dihydrocodeine tartrate, or IM morphine)	Post-operatively, continuous for 48h	Not mentioned	Bolus 0.5% Ropivacaine solution, then continuous 0.25% Ropivacaine 0.1 mL/kg/h for 48h	NRS	IV PCA + RA vs FICB + RA	Continuous FICB is a safe and effective technique for post-operative analgesia after hip fracture surgery.
Bang et al. (2016) [32]	RCT	n = 21 (10/11)	81.8	Spinal	IV ketorolac + IV fentanyl + IV PCA (fentanyl) + PO celecoxib BD 7 days + RA (IV tramadol)	Post-operatively, single shot	US-guided	40mL of 0.2% Ropivacaine with epinephrine	VAS	IV PCA + RA + Regular Analgesia vs FICB + IV PCA + RA + Regular Analgesia	FICB had significant opioid-sparing effect in the first 24h in hemiarthroplasty.
Ma et al. (2018) [31]	RCT	n = 88 (44/44)	83.89	Spinal	Pre-operative tramadol and paracetamol + IV PCA sufentanil post-operatively	Pre-operatively, continuous (from admission until surgery)	US-guided	Bolus 50mL 0.4% Ropivacaine + 0.2% ropivacaine 5mL/h	VAS	Regular analgesia + IV PCA vs Regular analgesia + FICB + IV PCA	US-guided FICB may provide a superior analgesia for elderly patients with hip fractures during the pre-operative period.
Hao et al. (2019) [23]	RCT	n = 85 (42/43)	72.41	Spinal	SHAM block with continuous 0.9% NaCl + RA (IM fentanyl)	Pre-operatively, continuous (unknown timing)	US-guided	Bolus 30mL 0.45% Ropivacaine + 0.25% Ropivacaine / 0.9% NaCl 6mL/h	VAS	SHAM + RA vs FICB + RA	Pre-emptive analgesia with continuous FICB is an effective technique to reduce pre-operative pain, opioid requirement, and post-operative delirium.
Hao et al. (2021) [27]	RCT	n = 120 (60/60)	76.355	Spinal	PO celecoxib on admission and 6h before surgery + IM parecoxib for 3 days post-operatively then to PO celecoxib + RA (morphine)	Intra-operatively (post-spinal), single shot	Landmark	Total of 30mL of 0.33% Ropivacaine Hydrochloride, 1% Lidocaine, and 5mg Dexamethasone	VAS	Regular analgesia + RA vs FICB + regular analgesia + RA	FICB provides superior post-operative short-term analgesic effect both at rest and with movement and accelerates post-operative short-term hip joint function recovery.
Dai et al. (2024) [29]	RCT	n = 62 (31/31)	75.325	GA	Flurbiprofen 1-2mg/kg + RA (tramadol or flurbiprofen)	Pre-operatively, single shot	US-guided	30mL of 0.33% Ropivacaine	VAS	RA vs FICB + RA	US-guided FICB combined with GA during hip fracture surgery has demonstrated efficacy in providing analgesia.
Caliskan et al. (2025) [30]	RCT	n = 50 (25/25)	79.5	Spinal	Paracetamol, tenoxicam + RA (IV tramadol 1mg/kg PRQDS)	Post-operatively, single shot	US-guided	30mL of 0.375% Bupivacaine	NRS	Regular analgesia + RA vs FICB + regular analgesia + RA	FICB is the preferable choice to ensure efficient analgesia with minimum analgesic consumption during first 24h post-operatively.
Garlich et al. (2020) [24]	Observational study	n = 725 (633/92)	84.25	Unknown	All pain medications up to primary medical team	Pre-operatively, either single shot or continuous (until POD1)	US-guided	Single shot - 30-40mL 0.25% Bupivacaine with Epinephrine, Continuous - 10-20mL bolus of 0.2% Bupivacaine + 0.2% Bupivacaine 6mL/h	VAS	Regular Analgesia vs FICB + Regular Analgesia	FICB reduces pre-operative opioid intake and has low rates of opioid-related adverse events.
Wang et al. (2021) [25]	Observational study	n = 31 (15/16)	79.65	GA	IV fentanyl post-operatively + IV PCA fentanyl)	Pre-operatively, single shot	US-guided	20 mL 0.375% Ropivacaine	VAS	IV PCA vs FICB + IV PCA	US-guided FICB in elderly undergoing total hip arthroplasty might accelerate the recovery after GA and relieve early post-operative pain.
Salottolo et al. (2022) [26]	Observational study	n = 517 (136/381)	76	Both GA and spinal	Acetaminophen OR hydrocodone with acetaminophen OR PO morphine OR IV opioids based on pain scores	Pre-/post-operatively, single shot or continuous	US-guided	Bupivacaine OR Ropivacaine OR Bupivacaine Liposomal	NRS	Regular Analgesia vs FICB + Regular Analgesia	FICB was not more effective than systemic analgesics for delirium, opioid consumption, or complications, but pain scores were significantly improved with FICB.
Bali and Ozmete (2023) [28]	Observational study	n = 41 (19/22)	85.705	GA	Paracetamol at closure + IV PCA (fentanyl) + RA (IV tramadol)	Intra-operatively, single shot	US-guided	40mL of 0.25% Bupivacaine	VAS	IV PCA + RA vs FICB + IV PCA + RA	FICB has a significant opioid-sparing effect and thus reduces opioid-related side effects in the first 24h after hip surgery.

Risk of Bias and Certainty Assessments

**Figure 2:**
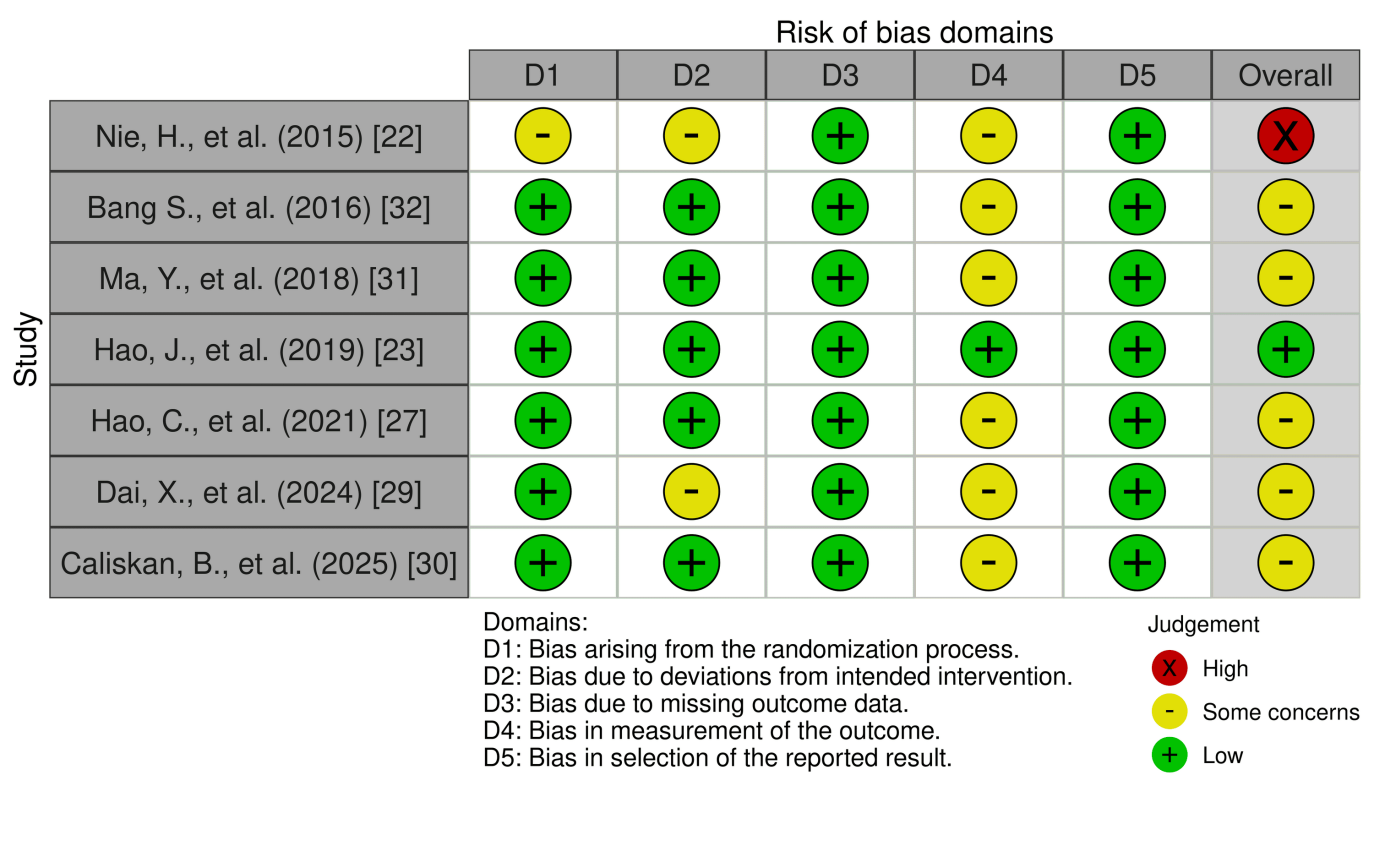
Cochrane Risk of Bias 2 (RoB2) results Studies: Nie et al. (2015) [22], Bang et al. (2016) [32], Ma et al. (2018) [31], Hao et al. (2019) [23], Hao et al. (2021) [27], Dai et al. (2024) [29], Caliskan et al. (2025) [30]

**Figure 3:**
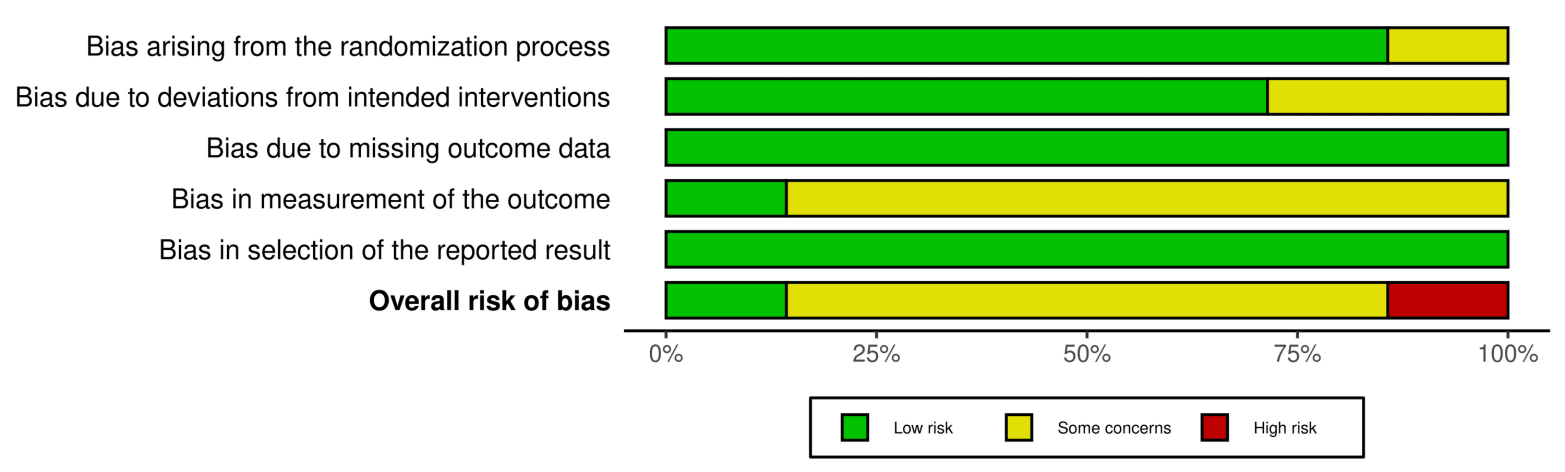
Cochrane Risk of Bias 2 results continued

The RoB2-derived risk of bias of the seven RCTs were varied, with five RCTs (n =5, 71.4%) [27,29-32] obtaining a ‘yellow/some concern’ risk-of-bias rating, one RCT [23] obtaining a ‘green/low’ risk-of-bias rating, and one RCT [22] obtaining a ‘red/high’ risk-of-bias rating. A recurring source of down-rating in the RoB2 tool that most (n =6, 85.7%) of the RCTs [22,27,29-32] succumbed to was in domain 4 (bias in measurement of the outcome), specifically signalling question 4.3, which those RCTs had failed due to the lack of participant blinding. The reason for the lack of blinding in those studies was ethical considerations withholding them from inserting a sham/placebo block just for the sake of blinding. The only RCT [22] that received a ‘red/high’ final risk-of-bias rating was a study that yielded ‘some concern’ in three of the five RoB2 domains: due to a lack of information on their randomisation process, a lack of participant blinding, and unclear use of appropriate analysis to estimate the effect of assignment to the intervention. The only RCT [23] which received a ‘green/low’ final risk-of-bias rating was the study that inserted a sham/placebo block, enabling true double blinding. Lastly, no single domain in any of the RCTs was in and of itself assigned a ‘high’ risk of bias. All RoB2 results are visualised using the RobVis tool in Figures 2, 3 [33].

All the four NRSIs that underwent the NOS risk-of-bias tool [24-26,28] achieved a ‘low’ risk-of-bias rating as per the NOS rating scale, receiving the top score of nine stars on the scale. It is also worthwhile to note that the NOS tool had been regarded as being too vague in certain aspects, leading to low inter-rater reliability. Hence, the risk-of-bias assessment by the NOS should also be taken with the proverbial pinch of salt [34]. All NOS results are detailed in Table 3.

**Table 3:** Newcastle-Ottawa Quality Assessment Scale for the included observational studies The asterisks are the rating scale used for the Newcastle-Ottawa Scale, It is a star system used to rate the risk of bias. One star corresponds to one asterisk, and two stars correspond to two asterisks.

Authors (year)	Study type	Selection				Comparability	Outcome			Final score	Quality	Risk of bias
		Representativeness of the exposed cohort	Selection of the non-exposed cohort	Ascertainment of exposure	Demonstration that outcome of interest was not present at start of the study	Comparability of cohorts on the basis of the design or analysis	Assessment of outcome	Was follow-up long enough for outcomes to occur	Adequacy of follow-up of cohorts			
Garlich et al. (2020) [24]	Observational study	*	*	*	*	**	*	*	*	9/9	High	Low
Wang et al. (2021) [25]	Observational study	*	*	*	*	**	*	*	*	9/9	High	Low
Salottolo et al. (2022) [26]	Observational study	*	*	*	*	**	*	*	*	9/9	High	Low
Bali and Ozmete (2023) [28]	Observational study	*	*	*	*	**	*	*	*	9/9	High	Low

With regards to the certainty of evidence assessment applied to the aforementioned five pre-determined outcomes, the GRADE tool revealed that evidence regarding day one post-operative pain scores at rest was judged to be of moderate certainty, evidence regarding day one post-operative pain scores on movement was judged to be of moderate certainty, evidence regarding post-operative incidence of delirium was judged to be of very low certainty, evidence regarding day one post-operative opioid consumption was judged to be of high certainty, and evidence regarding average LOS was judged to be of moderate certainty.

The mix of study types (RCTs and NRSIs) in our review presented a dilemma with regards to how our GRADE assessment should be performed. In response, we decided to adopt suggestions by the U.S. Centers for Disease Control and Prevention Advisory Committee on Immunization Practices (US CDC ACIP) to first utilise RCTs in our GRADE assessments due to their higher inherent quality and to only include the relevant NRSIs in the decision process if the initial GRADE result for the RCTs alone in that particular outcome measure fell to a ‘low’ or a ‘very low’ certainty, in an attempt to better support the RCTs with complementary evidence and data, as NRSI evidence certainty would initially start at a ‘low’ grade regardless [35]. The only outcome that warranted such inclusion of NRSIs was the ‘post-operative delirium incidence’ outcome, as it initially fell to a ‘very low’ certainty score when only RCTs were involved - firstly due to inconsistencies in study findings amongst the three relevant RCTs [22,23,29] (two [23,29] suggested that the control/CSA groups had a higher incidence of delirium when compared to the intervention/FICB group, whilst one [22] suggested the opposite), secondly due to the inherent risk of bias in the assessment of delirium by human outcome assessors (further explained below), and lastly due to imprecision secondary to a relatively small outcome-specific sample size (n =251). Notably, after the inclusion of two relevant NRSIs [24,26], the imprecision concern regarding the small sample size was resolved with an addition of another 1,242 patients, but further concern developed regarding the inconsistencies (now there were three studies [22,24,26] with a total of 1,346 patients, suggesting that the incidence of delirium was higher in the intervention/FICB groups when compared to the control/CSA groups, and two studies [23,29] with a total of 147 patients suggesting otherwise, although the sample size discrepancy between both directions of this effect should be noted) led to the maintained judgement of a ‘very low’ certainty score for this outcome. The other three non-low certainty outcomes were rated to be of moderate certainty, consisting of the static and dynamic post-operative pain outcomes, both being rated as such due to the risk of bias associated with their RCTs’ lack of blinding, and the ‘average length of stay’ outcome, being rated as such due to the imprecision secondary to its relatively small, albeit consistent, sample size (n = 182) from two relevant RCTs [27,29]. The reviewers found no other significant concerns raised in all studies to satisfactorily warrant a further downgrade in any of the GRADE domains apart from those mentioned above. Our GRADE outcomes and results are illustrated in Table 4.

**Table 4:** Summary of findings Patients or population: patients ≥ 70 years old presenting with hip fractures that require surgical management Settings: Inpatient surgical Intervention: FICB Comparison: routine systemic analgesia *GRADE Working Group grades of evidence: High = This research provides a very good indication of the likely effect. The likelihood that the effect will be substantially different is low. Moderate = This research provides a good indication of the likely effect. The likelihood that the effect will be substantially different is moderate. Low = This research provides some indication of the likely effect. However, the likelihood that it will be substantially different is high. Very low = This research does not provide a reliable indication of the likely effect. The likelihood that the effect will be substantially different is very high. Substantially different = A large enough difference that it might affect a decision. FICB, fascia iliaca compartment block; NRSI, non-randomised study of intervention; RCT, randomised controlled trial

Outcomes	Impact	Number of participants (studies)	Certainty of the evidence (GRADE)*
Post-operative pain at rest at POD1	A difference in static pain outcome affecting patient comfort could be seen with the addition of FICB to current analgesic standards.	530 (7 RCTs)	⊕⊕⊕⊖ Moderate
Post-operative pain on movement at POD1	A difference in dynamic pain outcome affecting early rehabilitation could be seen with the addition of FICB to current analgesic standards.	208 (2 RCTs)	⊕⊕⊕⊖ Moderate
Post-operative delirium	The addition of FICB could influence the incidence of post-operative delirium, which might be related to pain or other morbidities.	1,493 (3 RCTs + 2 NRSIs)	⊕⊖⊖⊖ Very Low
Post-operative opioid consumption at POD1	FICB can affect post-operative opioid consumption rates and consequently the adverse side effects of it.	322 (5 RCTs)	⊕⊕⊕⊕ High
Average length of stay	The implementation of FICB could influence the average length of stay for such patients, potentially affecting treatment costs and patient well-being.	182 (2 RCTs)	⊕⊕⊕⊖ Moderate

Regarding the five relevant studies [22-24,26,29] in our delirium outcome measure, four used the Confusion Assessment Method (CAM), whilst one [29] used the Mini-Mental State Examination (MMSE) and the CAM. We felt the need to attribute an inherent risk of bias to the assessment of delirium due to previously published studies surrounding the subpar case-finding confirmatory ability of the MMSE in delirium, alongside staff tendency towards erroneous retrospective use of the assessment tools, the underreporting tendency observed when using the CAM on patients with a reduced consciousness level, and the difficulties in achieving sufficient user training for these tools facilitating the accurate differentiation of delirium from dementia, which is a common pitfall in such confusion assessments amongst healthcare professionals [36-38].

Primary Outcomes and Subgroup Analyses

The primary outcome (post-operative pain) findings were first split into static and dynamic groups. These two groups were later each split into VAS and NRS groups due to a previous study demonstrating poor agreement between these two scales in the older population [16]. We managed to obtain many different data points for the aforementioned groups, across a variety of time points (from pre-operative/pre-block measurements, to measurements at 1, 2, 3, 4, 6, 8, 12 hours post-operatively, to 1, 2, 3 days post-operatively). All studies included different combinations of time points in their measurements, with some studies having more measurements than others, and some studies lacking pre-operative time points. Given the widely heterogeneous data pool, we best endeavoured to summarise trends and values in this section. For the purposes of the rest of this section, data will be presented as ‘mean’ (n = number of contributing studies, SD = standard deviation). Our primary outcome and its respective subgroup analyses are detailed below. Tables 5, 6 illustrate the collected static and dynamic pain score data detailed in this section.

**Table 5:** Pain outcomes at rest (static) ND, no data; PO, post-operative; POD, post-operative day

Author (year)	Pain score	Pre-operative (control)	Pre-operative (FICB)	PO1hr (control)	PO1hr (FICB)	PO2hr (control)	PO2hr (FICB)	PO3hr (control)	PO3hr (FICB)	PO4hr (control)	PO4hr (FICB)	PO6hr (control)	PO6hr (FICB)	PO8hr (control)	PO8hr (FICB)	PO12hr (control)	PO12hr (FICB)	POD1 (control)	POD1 (FICB)	POD2 (control)	POD2 (FICB)	POD3 (control)	POD3 (FICB)
Bang et al. (2016) [32]	VAS	ND	ND	ND	ND	ND	ND	ND	ND	2	2.5	ND	ND	2.6	2.7	2.7	2.8	2.5	2.6	2.6	3	ND	ND
Ma et al. (2018) [31]	VAS	4.7	4.4	ND	ND	ND	ND	ND	ND	ND	ND	ND	ND	ND	ND	ND	ND	1.75	2	1.6	1.85	ND	ND
Hao et al. (2019) [23]	VAS	8.07	7.81	0.8	0.74	ND	ND	ND	ND	ND	ND	ND	ND	ND	ND	ND	ND	1.19	1.15	1.05	1	0.87	0.84
Hao et al. (2021) [27]	VAS	4.92	5	ND	ND	ND	ND	ND	ND	ND	ND	4.53	3.02	ND	ND	ND	ND	4.2	2.86	ND	ND	3.44	2.73
Dai et al. (2024) [29]	VAS	ND	ND	ND	ND	ND	ND	ND	ND	ND	ND	3	2	ND	ND	ND	ND	4	3	ND	ND	ND	ND
Garlich et al. (2020) [24]	VAS	4	4.2	ND	ND	ND	ND	ND	ND	ND	ND	ND	ND	ND	ND	ND	ND	2.7	3.1	2.5	3	ND	ND
Wang et al. (2021) [25]	VAS	ND	ND	ND	ND	ND	ND	ND	ND	ND	ND	ND	ND	ND	ND	ND	ND	3.6	2.7	3.4	3	ND	ND
Bali and Ozmete (2023) [28]	VAS	ND	ND	4.8	0.4	ND	ND	3.8	0.85	ND	ND	3.75	2.25	ND	ND	3.4	3	3	2.5	ND	ND	ND	ND
Nie et al. (2015) [22]	NRS	ND	ND	ND	ND	1.15	0.65	ND	ND	1.2	0.65	2.225	1.625	ND	ND	1.7	1.625	1.8	1.1	1.5	0.675	ND	ND
Caliskan et al. (2025) [30]	NRS	6.8	7.15	ND	ND	ND	ND	ND	ND	5.2	2.2	ND	ND	5.4	4.15	5.4	3.15	3.95	2.5	ND	ND	ND	ND
Salottolo et al. (2022) [26]	NRS	4.7	3.9	ND	ND	ND	ND	ND	ND	ND	ND	ND	ND	ND	ND	ND	ND	3.5	2.9	ND	ND	ND	ND

**Table 6:** Pain outcomes on movement (dynamic) ND, no data; PO, post-operative; POD, post-operative day

Author (year)	Pain score	Pre-operative (control)	Pre-operative (FICB)	PO1hr (control)	PO1hr (FICB)	PO3hr (control)	PO3hr (FICB)	PO6hr (control)	PO6hr (FICB)	PO12hr (control)	PO12hr (FICB)	POD1 (control)	POD1 (FICB)	POD2 (control)	POD2 (FICB)	POD3 (control)	POD3 (FICB)
Ma et al. (2018) [31]	VAS	7.2	7.3	ND	ND	ND	ND	ND	ND	ND	ND	3.35	3.3	2.85	3.05	ND	ND
Hao et al. (2021) [27]	VAS	ND	ND	ND	ND	ND	ND	6.2	4.17	ND	ND	5.53	4.02	ND	ND	4.47	3.54
Wang et al. (2021) [25]	VAS	ND	ND	ND	ND	ND	ND	ND	ND	ND	ND	4.9	3.4	4.2	3.4	ND	ND
Bali and Ozmete (2023) [28]	VAS	ND	ND	5.58	0.75	5	1.6	4.45	3.6	4.2	3.7	3.7	3.2	ND	ND	ND	ND

For the static pain + control/CSA group, six studies reported pre-operative/pre-block measurements, with four [23,24,27,31] reporting via the VAS scale and two [26,30] reporting via the NRS scale. The mean control group pre-operative static pain scores were 5.42 (n = 4, SD = 1.57) per VAS and 5.75 (n = 2, SD = 1.05) per NRS. The most commonly measured post-operative time point in the control group was at post-operative day one (POD1), with all studies (n = 11) doing so, providing mean control group POD1 static pain scores of 2.87 per VAS (n = 8, SD = 0.99) and 3.08 per NRS (n = 3, SD = 0.93). The second most commonly measured post-operative time point in the control group was at post-operative day two (POD2), with six studies doing so, providing mean control group POD2 static pain scores of 2.23 per VAS (n = 5, SD = 0.82) and 1.5 per NRS (n = 1, SD = n/a).

For the static pain + intervention/FICB group, six studies reported pre-operative/pre-block measurements, with four [23,24,27,31] reporting via the VAS scale and two [26,30] reporting via the NRS scale. The mean FICB group pre-operative static pain scores were 5.35 per VAS (n = 4, SD = 1.45) and 5.53 per NRS (n = 2, SD = 1.63). The most commonly measured post-operative time point in the FICB group was at POD1, with all studies (n = 11) doing so, providing mean FICB group POD1 static pain scores of 2.49 per VAS (n = 8, SD = 0.60) and 2.17 per NRS (n = 3, SD = 0.77). The second most commonly measured post-operative time point in the FICB group was at POD2, with six studies doing so, providing mean FICB group POD2 static pain scores of 2.37 per VAS (n = 5, SD = 0.82) and 0.68 per NRS (n = 1, SD = n/a).

**Figure 4:**
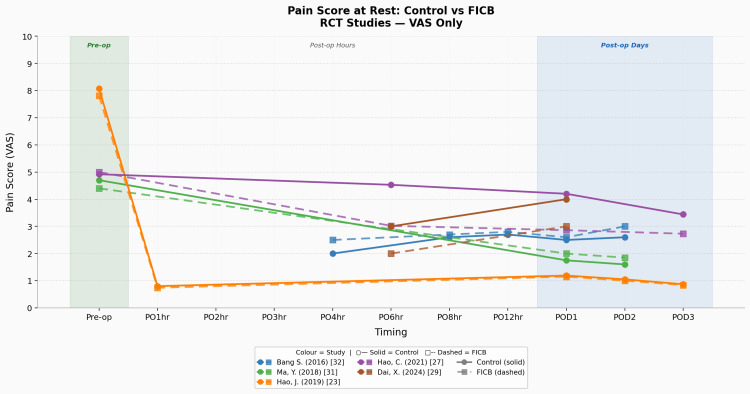
Pain score at rest: control vs FICB (RCT studies using VAS only) Studies: Bang et al. (2016) [32], Ma et al. (2018) [31], Hao et al. (2019) [23], Hao et al. (2021) [27], Dai et al. (2024) [29] FICB, fascia-iliaca compartment block; RCT, randomised controlled trial; VAS, visual analogue scale

**Figure 5:**
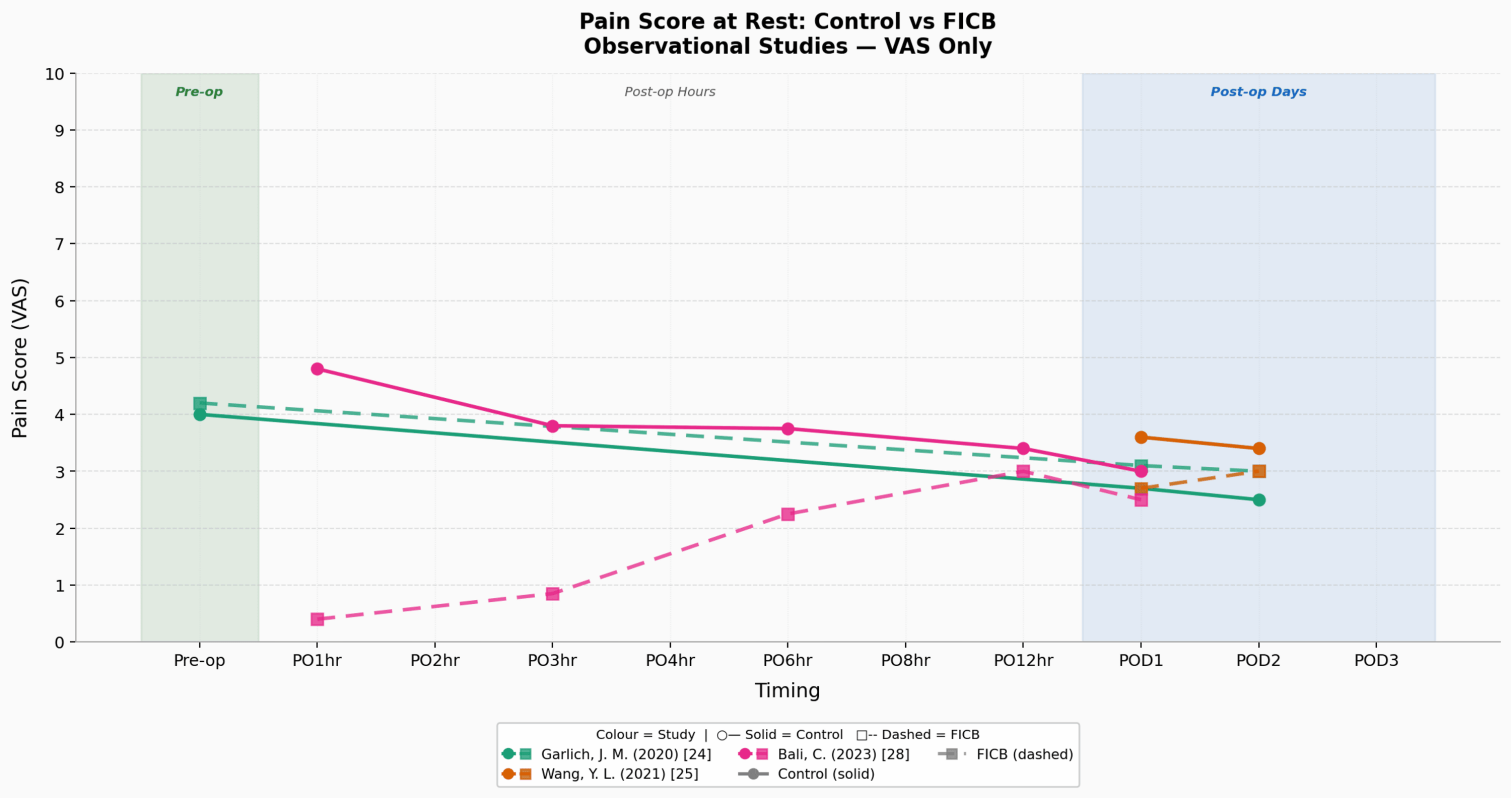
Pain score at rest: control vs FICB (observational studies using VAS only) Studies: Garlich et al. (2020) [24], Wang et al. (2021) [25], Bali and Ozmete (2023) [28] FICB, fascia-iliaca compartment block; VAS, visual analogue scale

**Figure 6:**
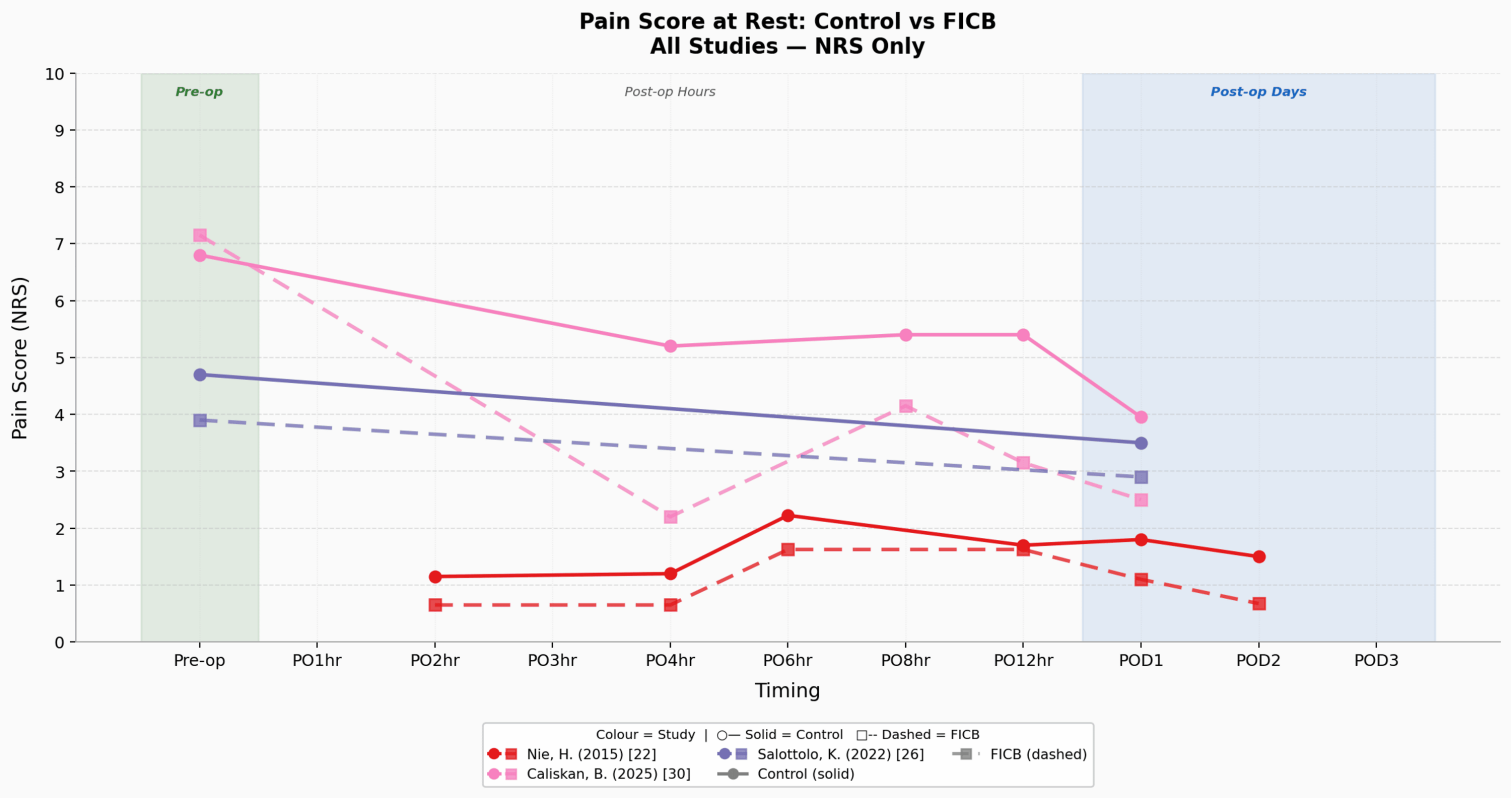
Pain score at rest: control vs FICB (all studies - NRS only) Studies: Nie et al. (2015) [22], Caliskan et al. (2025) [30], Salottolo et al. (2022) [26] FICB, fascia-iliaca compartment block; NRS, numerical rating scale; RCT, randomised controlled trial

Static pain graphs separated into various assessment tools and study types illustrating the trends and patterns detailed in this section can also be found in Figures 4-6.

For the dynamic pain + control/CSA group, there was only one study [31] reporting a pre-operative pain score. It was performed via VAS and resulted in a mean control group pre-operative dynamic VAS score of 7.2 (n = 1, SD = n/a). The most commonly measured post-operative time point in the control group was at POD1, with four studies doing so, all of which used VAS, providing a mean control group POD1 dynamic VAS score of 4.37 (n = 4, SD = 0.88). The second most commonly measured post-operative time point in the control group was at POD2, with two studies doing so, all of which used VAS, providing a mean control group POD2 dynamic VAS score of 3.53 (n = 2, SD = 0.68).

For the dynamic pain + intervention/FICB group, there was only one study [31] reporting a pre-operative pain score. It was performed via VAS and resulted in a mean FICB group pre-operative dynamic VAS score of 7.3 (n = 1, SD = n/a). The most commonly measured post-operative time point in the FICB group was at POD1, with four studies doing so, all of which used VAS, providing a mean FICB group POD1 dynamic VAS score of 3.48 (n = 4, SD = 0.32). The second most commonly measured post-operative time point in the FICB group was at POD2, with two studies doing so, all of which used VAS, providing a mean FICB group POD2 dynamic VAS score of 3.23 (n = 2, SD = 0.18).

**Figure 7:**
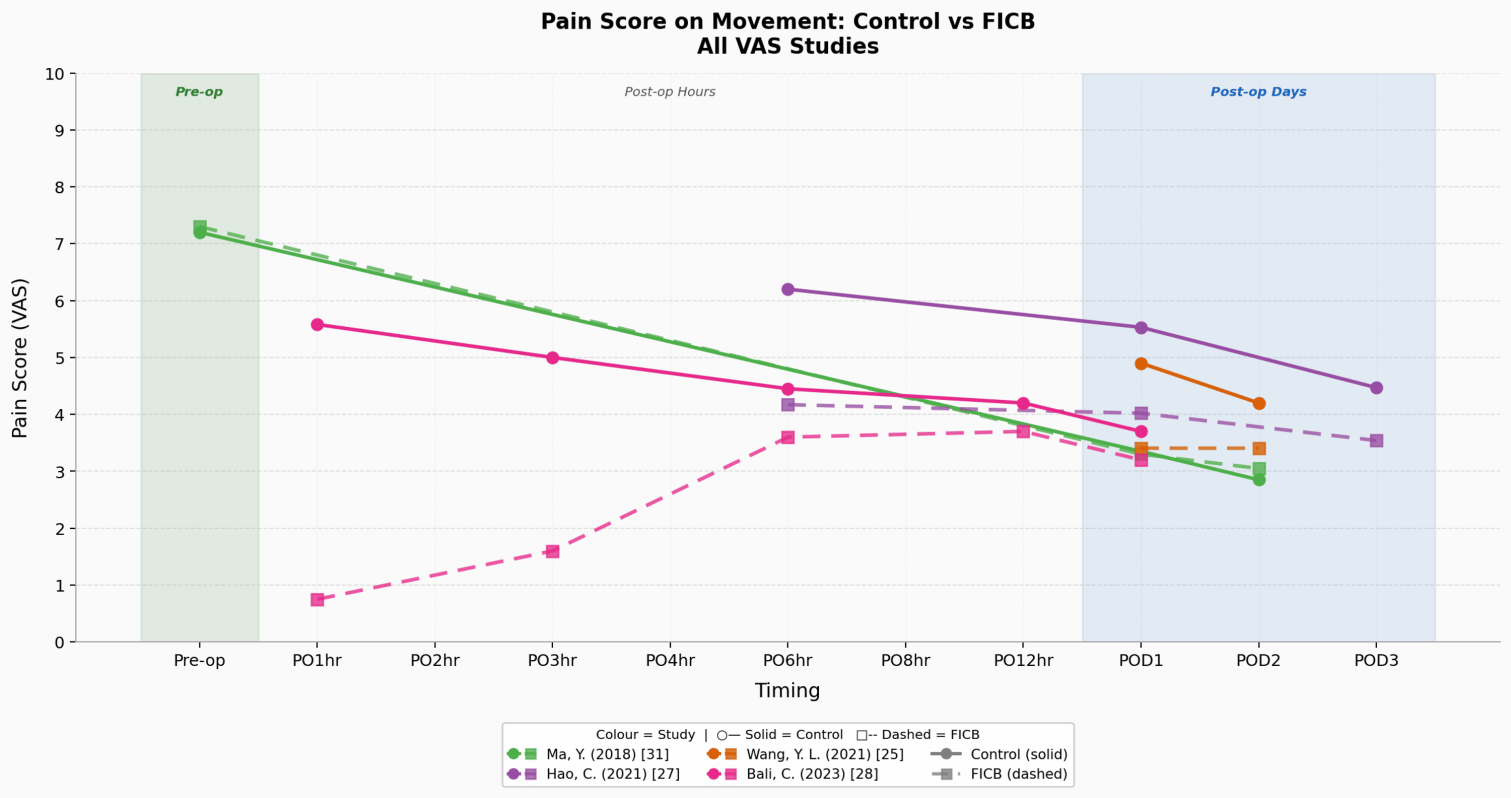
Pain score on movement: control vs FICB (all studies included - notably only VAS data available) Studies: Ma et al. (2018) [31], Hao et al. (2021) [27], Wang et al. (2021) [25], Bali and Ozmete (2023) [28] FICB, fascia-iliaca compartment block; VAS, visual analogue scale

Figure 7 shows a dynamic pain graph illustrating the trends and patterns detailed in this section. No NRS data were available for this outcome.

The average static pain intensity difference (Δ static pain) in the control group (CG) between POD1 and pre-operative levels was as follows: CG Δ static pain of -2.55 per VAS and -2.67 per NRS. For the FICB group, it was as follows: FICB Δ static pain of -2.86 per VAS and -3.36 per NRS. The key result here is that the FICB group has a consistently wider Δ static pain (larger decrease) across both the VAS and NRS when compared to the control/CSA group.

The average dynamic pain intensity difference (Δ dynamic pain) in the control group (CG) between POD1 and pre-operative levels was as follows: CG Δ dynamic pain of -2.83 per VAS. For the FICB group, it was as follows: FICB Δ dynamic pain of -3.82 per VAS. The key result here is that the FICB group has a wider Δ dynamic pain VAS (larger decrease) when compared to the control/CSA group. There was no applicable NRS data for this analysis.

To conclude the primary outcome (pain score) results. There is an objectively reproducible peri-operative reduction in static and dynamic pain amongst both the CSA and FICB groups, and it is more so apparent in the FICB group, suggesting a greater pain reduction ability in the FICB when compared to CSA for this population demographic. This is underscored by the FICB’s consistently greater reduction in pain scores amongst the VAS and NRS metrics, across multiple study locations, study types, sample sizes, and hip fracture surgical procedures. Evidence surrounding these two pain outcomes was of ‘moderate’ certainty, as per GRADE.

Secondary Outcomes and Subgroup Analyses

Our secondary outcome measures included the incidence of post-operative delirium, average LOS, and post-operative opioid consumption. The certainty of evidence towards each outcome was varied and will be stated at each outcome conclusion. For the purposes of the rest of this section, data will be presented as ‘mean percentage’ (n = number of contributing studies, SD = standard deviation). The secondary outcome measures are presented in Table 7.

**Table 7:** Summary of secondary outcome measures LOS, length of hospital stay; MME, morphine milligram equivalent; PONV, post-operative nausea and vomiting

Measures	Post-operative delirium	LOS	Post-operative opioid consumption (MME)	PONV/dizziness	Pruritus
Control	136±43.72 out of 895 (15.2%, n=5)	7.60±5.19 (n=5)	34.9±16.6mg (n=8)	41±6.53 out of 157 (26.1%, n=5)	9±2 out of 82 (11.0%, n=3)
FICB	67±9.91 out of 598 (11.2%, n=5)	7.17±4.34 (n=5)	19.7±10.1mg (n=8)	20±5.70 out of 160 (12.5%, n=5)	1±0.58 out of 84 (1.2%, n=3)

Incidence of post-operative delirium was measured in five studies totalling 1,493 participants, of which three were RCTs [22,23,29] and two were NRSIs [24,26]. The incidence of delirium when measured in percentage of study arm sample size (%) was 15.2% (136/895, n = 5, SD = 43.72) in the control/CSA group, whilst it was 11.2% (67/598, n = 5, SD = 9.91) in the intervention/FICB group. The greatest incidence of delirium in any singular study [23] was 35.7% in a control arm, whilst it was 19.6% in the FICB arm [22]. The lowest incidence of delirium in any singular study [26] was 4.4% in a control arm, whilst it was 7.6% in the FICB arm [26]. The evidence surrounding post-operative delirium in our review was judged to be of ‘very low’ certainty, as per GRADE.

**Figure 8:**
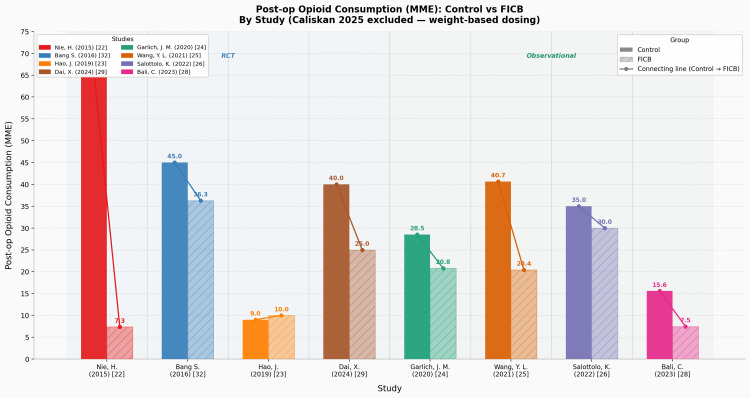
Post-operative opioid consumption in MME: control vs FICB, by study Studies: Nie et al. (2015) [22], Bang et al. (2016) [32], Hao et al. (2019) [23], Dai et al. (2024) [29], Garlich et al. (2020) [24], Wang et al. (2021) [25], Salottolo et al. (2022) [26], Bali and Ozmete (2023) [28] FICB, fascia iliaca compartment block; MME, morphine milligram equivalent

Next, the mean LOS measured in days was derived from five studies totalling 1,465 participants, of which two were RCTs [27,29] and three were NRSIs [24,26,28], and was found to be 7.60 (n = 5, SD = 5.19) in the control/CSA group and 7.17 (n = 5, SD = 4.34) in the intervention/FICB group. A notable outlier was an RCT in China [27] having an average LOS of 17.98 days in their control group and 15.86 days in their FICB group. All other included studies had identical LOS (five days) for both their control and FICB groups. The evidence surrounding LOS in our review was judged to be of ‘moderate’ certainty, as per GRADE. Lastly, post-operative opioid consumption was measured in nine studies involving 1,636 participants, of which five were RCTs [22,23,29,30,32] and four were NRSIs [24-26,28]. All measures of consumption were converted appropriately to morphine milligram equivalent (MME) for standardisation. One of the studies [30] did not provide the body weight data required to calculate MME; hence, all related calculations excluded the data from that study. The mean MME in post-operative opioid consumption for the control group was 34.9mg (n = 8, SD = 16.6mg), whilst for the FICB group, it was 19.7mg (n = 8, SD = 10.1mg). The greatest reduction in opioid consumption when comparing between the FICB and control group of any singular study [22] was by 58.5mg MME (an 89% reduction in the FICB group), whilst the smallest reduction [24] seen was by 5mg MME (a 14% reduction in the FICB group). Only one study [23] showed an increase in MME consumption post-operatively, by 1mg MME (an 11% increase). The evidence surrounding post-operative opioid consumption in our review was judged to be of ‘high’ certainty, as per GRADE. This breakdown of post-operative opioid consumption in the relevant individual studies is intuitively displayed for easy visualisation in Figure 8.

Apart from the data we aimed to capture, we also managed to unexpectedly extract data with regards to incidences of post-operative nausea and vomiting (PONV), dizziness, and pruritus. The general consensus suggested from these findings was that FICB use led to a lower incidence of PONV/dizziness (FICB: 12.5% incidence amongst 160 patients; control: 26.1% incidence amongst 157 patients) and pruritus (FICB: 1.2% incidence amongst 84 patients / control: 11% incidence amongst 82 patients). We did not expand on the details of these findings as they were not part of our review outcomes, although these data do provide further reassurance (albeit of unknown evidence certainty) for the use of FICB in these patients.

In conclusion, regarding our secondary outcomes, our review suggests that the FICB causes no significant differences in direction or magnitude of effect toward the post-operative incidence of delirium in patients of this demographic when compared to patients using CSA, albeit the ‘very low’ certainty of evidence for it. Next, it also suggests that the FICB causes no significant changes in the average LOS for patients of this demographic when compared to patients using CSA, where this is supported by ‘moderate’ certainty evidence. Lastly, it suggests with ‘high’ certainty evidence that the FICB has a significant opioid-sparing effect on patients during the post-operative period, and this effect is more apparent in the FICB group than the CSA group.

Discussion

Confident, evidence-based findings will form the backbone of clinical practice recommendations, leading to standardised care that targets the best possible outcomes after thorough risk-benefit analysis. Therefore, this review aims to contribute to the advent of an appropriate, evidenced-based regional block technique, which we expect will bring increased patient satisfaction and improved outcomes to a population subset as frail and vulnerable as our target population (elderly patients ≥ 70 years old with hip fractures). Our final inclusion of 11 studies (seven RCTs and four NRSIs) [22-32], totalling 1,844 participants with a mean age of 78.7 years, is a meaningful and clinically relevant sample with regards to our topic. In addition, our included studies maintain a satisfactory geographical spread (involving China, Turkey, the USA, and Korea), which suggests a good variability. It is also apparent that all 11 studies stand by the FICB, with all of them agreeing in support of the FICB over CSA in pain outcomes and its opioid-sparing effect. Importantly, the heterogeneity in study design that we experienced, including differing anaesthesia/analgesia types, FICB modalities, pain scales, and opioid uses, adds complexity to any attempt at a direct comparison, although it also does reflect real-world practices.

Pain at Rest

The apparent direction of effect suggests that although both patient groups showed meaningful reductions in static pain across their peri-operative time frame, which is expected given the use of surgical intervention and multimodal analgesia, the important comparison is instead the magnitude of that effect. When looking at the overall Δ static pain VAS/NRS and the Δ dynamic pain VAS between both groups, it consistently demonstrates the superior analgesic ability of the FICB. It is also important to note the trend seen in the graphs above, wherein there is an occasionally observed relative increase/plateau in pain scores from the immediate post-operative period to the 24/48-hour post-operative mark. This 24-hour mark is especially important, as the POD1 time point was universally reported in all 11 studies, not just making it the most robust comparative datapoint in the review but also the most clinically significant datapoint, given that post-operative pain is more intense the closer you are from completion of the surgery and the fact that early mobilisation (facilitated by improved pain control) increases the odds of earlier discharge/reduced LOS [39]. This trend could be attributed to the block wearing off during the 24-hour time frame, given that most studies administered this block in a single-shot pre/intra-operative format. In addition, a comparative study using ultrasound-guided peripheral nerve blocks for elective limb surgeries has shown that a standardised amount of 20 mL of 0.5% bupivacaine and 0.5% ropivacaine each have a sensory block duration (minutes) of 472.5±60.3 min and 390.6±54.2 min, respectively [40]. Interestingly, we had one study [22] that provided a continuous post-operative 48-hour long FICB and that study exhibited the largest difference in post-operative opioid consumption when comparing each FICB and control group of every single study. That same study also reported the lowest FICB group/overall pain scores amongst all studies in the NRS study subgroup, underscoring the efficacy of the continuous FICB infusion method post-operatively for static pain. The only limitation of static pain measurements noted in this study was the inconsistent time points chosen by the different studies, leading to ‘crowding’ of data in certain ‘commonly measured’ time points, which then manifests itself in the form of graphs having inconsistent start/end points, and ‘data/plotting gaps’ in certain time windows, which could consequently lead to a possible misrepresentation of actual trend.

Pain on Movement

Dynamic pain data were considerably sparser than static pain data, as only four studies [25,27,28,31] contributed to POD1 dynamic VAS. Since all studies used only the VAS, it limits the breadth of conclusions that we can make. The dynamic pain direction of effect remained consistent with what was observed in the static pain measures. Consistently lower pain scores across all measured time points were seen in the FICB groups when compared to the control groups across all but one study [31] measuring dynamic pain. The study in question provided a near-identical reporting of pain scores between both groups across all time points, as is demonstrated by both its lines on the graph being nearly superimposed as one. Better dynamic pain control, as suggested by the FICB results, may translate to earlier and more effective physiotherapy engagement, potentially improving rehabilitation outcomes and downstream functional recovery. A limitation of assessing dynamic pain would be the possible inconsistencies in methods used by assessors to evoke their definition of ‘dynamic’ pain (i.e. the movements that patients are asked to perform when assessing for dynamic pain), and certain studies reported their methods, whilst others did not. Another limitation would be the avoidance of dynamic pain testing by the patients, presumably out of fear of further injury, alongside other patient factors [41], leading to reduced sample sizes/outcome significance when compared to the abundance of static pain outcome data.

Post-Operative Delirium

The incidence of post-operative delirium was numerically lower in the FICB group (11.2%) versus the CSA group (15.2%), suggesting a potential protective effect of the FICB. Although the ‘very low’ certainty of evidence here is a reminder to remain cautious when interpreting these results, the fundamental certainty-degrading problem is regarding the inconsistency of evidence: three studies [22,24,26] suggested a higher delirium incidence in the FICB group, whilst two studies [23,29] suggested the opposite. The sample size discrepancy is obvious, although it needs to be said that much of the sample size in the larger group comes from the addition of two large NRSIs (which are perceived to be a lower tier of evidence when compared to RCTs) as per the rationale detailed in the GRADE Results section. Limitations here would include the fact that post-operative delirium is often complex and multifactorial, and it would be difficult to truly attribute any direction of effect to the addition of FICB to the analgesia plan [42], unless the magnitude of the effect, if any, was sufficiently profound. Other than that, there is also a complex interplay of causative factors with the manifestations of delirium - such as how uncontrolled post-operative pain can cause delirium, which can be worsened/improved with opioid consumption, and that delirium itself can lead to patients not asking for their ‘as required’ pain relief - leading to worsened pain control as well. Delirium can also develop at different post-operative time points, which raises the question as to when delirium should be assessed. A final limitation noted by the authors is regarding how pain reporting may be affected by patients developing delirium acutely after their operation, possibly through under/over-reporting of pain whilst in acute confusional states.

Opioid Consumption

The FICB group consumed substantially less opioids post-operatively on average: 19.7mg MME vs 34.9mg MME in the CSA group, which is a reduction of approximately 43.5%, a clinically significant result. This is substantially relevant clinically as the elderly population is naturally more susceptible to opioid adverse effects such as respiratory depression, constipation, urinary retention, and delirium. Hence, this finding, at its current ‘high’ level of GRADE evidence certainty, if implemented, will undoubtedly improve patient safety and outcomes. The conversion of the different analgesia types used in the various studies to MME is required for standardisation and should be continued and facilitated (through the provision of necessary data by original articles to calculate MME) by all future studies/trials/reviews. As such, it is worth noting the one study [30] that could not contribute to our MME analysis due to missing body weight data as a minor limitation. Another limitation we noted was the absence of specific standardised information from the relevant studies regarding their use of rescue/post-operative analgesia, wherein multiple studies simply reported the type of analgesia and dosages used, alongside indications for administration in the post-anaesthetic care unit but not the indications for use when back on the ward. One large, multicentre study [24] only mentioned that the use and type of post-operative analgesia was up to the discretion of the primary clinicians looking after the patients, leading to difficult integration of that potentially statistically useful study into our review in this regards. Although we recognise that introducing a widespread standardised guideline for use of rescue analgesia is a significant logistical challenge which is unlikely to be practically feasible, we suggest that improvements be made to its reporting, especially with regards to specific indications and frequencies of rescue/post-operative analgesia use once on the ward.

Average LOS

The idea of measuring average LOS was to hopefully determine if the FICB led to reduced LOS, as we hypothesised that its mechanisms include better pain control (leading to earlier discharge confidence), early mobilisation (leading to earlier ‘physiotherapy-safe’ outcomes and reduced venous-thromboembolism incidence which is a potentially terminal event in patients of this demographic), and reduced delirium (which could delay discharge). The average LOS was marginally shorter in the FICB group (7.17 days) vs the CSA group (7.60 days) - a difference of less than half a day on average. It is difficult to tell if this is statistically significant in this context. A significant outlier in LOS data, as seen in one of our studies from China [27], could be attributed to the possible differences in healthcare systems and discharge practices. Conversely, all other relevant studies that we included had reported identical LOS outcomes at five days on average, for which no obvious explanation could be deduced by the authors. The main limitation here would be the differences in hospital resources amongst study sites (i.e. a larger, relatively resource ‘rich’ hospital with greater physiotherapy manpower could begin mobilising patients sooner or provide twice-daily physiotherapy reviews of patients, leading to earlier rehabilitation and discharges, whereas a smaller hospital with less funding may encounter multiple barriers to discharge, such as reduced clinician or multi-disciplinary team member input which would inevitably worsen post-operative outcomes and consequently extend LOS, alongside increased incidences of post-operative complications due to reduced diagnostic and preventative abilities).

*Review* *Limitations and Recommendations*

Several limitations must be acknowledged. Limitations of our search include the lack of any librarian input toward the search strategy and the exclusion of articles without available English-language full-text translations. Next, the most pervasive methodological limitation across all included RCTs was the near-universal absence of participant blinding, which degraded evidence certainty by introducing a degree of bias into pain outcome measurements - albeit ethically justified as discussed in the Results section. Also, significant heterogeneity in study design, including variation in anaesthesia types, FICB timing, insertion technique, drug dosage, and pain measurement scales, limits our direct comparison between studies and precludes formal meta-analysis. There was also a noticeable geographical concentration of studies based in China (n = 6) [22,23,25,27,29,31], raising possible questions regarding the generalisation of our reported findings, though this can be counteracted by the focussed involvement of studies from other locations. Lastly, outcome-specific sample sizes for post-operative delirium and average LOS were relatively small, limiting statistical and conclusory power for those domains.

We also felt prudent to mention the PENG block as an emerging contender in the field, with a previous double-blinded RCT even showing improved efficacy when compared to the FICB, although it was also mentioned that more research on that comparison with larger sample sizes is required to further reinforce/refute that finding [43]. With regards to the FICB itself, future research should prioritise adequately powered, multi-centre RCTs that incorporate sham blocks or placebo for true blinding where ethically permissible, alongside standardised and prospectively applied control group analgesia classifications and FICB technique, especially regarding timing, insertion technique, drugs, and dosage. It may also be beneficial to look into functional recovery outcomes, including time to mobilisation or physiotherapy milestones. Going forward, we would recommend that a systematic review with a librarian-led search strategy and a corresponding statistician-led meta-analysis for this effect in this population group be pursued if adequately standardised data arises through newer studies and to build upon the directional consistency and effect precision demonstrated in this review. Lastly, given that the current research consensus already supports the use of the FICB with regards to pain outcomes (as demonstrated in this review), we would suggest for a shift in FICB research toward specifically determining the effect it has on other factors (such as focussing on the secondary outcomes in this review or comparing it to other regional block modalities such as the PENG block as mentioned above).

## Conclusions

This review demonstrated that the FICB, as part of a multimodal analgesia care plan, offers meaningful clinical benefits over CSA in elderly hip fracture patients requiring surgery. With moderate to high evidence certainty, FICB reduced both static and dynamic pain scores and, consequently, post-operative opioid consumption. Whilst a marginal reduction in average LOS was observed, clinical significance remains uncertain due to GRADE imprecision. Other secondary outcomes, such as post-operative delirium, PONV, and pruritus, remained inconclusive due to the very low evidence certainty and insufficient availability of evidence. The consistency of FICB-supportive findings and conclusions across all 11 studies provides a compelling pain-specific rationale for the routine incorporation of the FICB (ideally a continuous infusion, until 48 hours post-operatively) into multimodal analgesia protocols worldwide, especially for this vulnerable subset of population.

## References

[1] Wang Y, Tan L, Dong J, Zhang J, Zhu J, Zhang Y (2026). Global epidemiology of hip fractures in adults aged 70 years and older from 1990 to 2021: a cross-sectional analysis from Global Burden of Disease Study 2021 [Online ahead of print]. Chin J Traumatol.

[2] Marks R, Allegrante JP, MacKenzie CR, Lane JM (2003). Hip fractures among the elderly: causes, consequences and control. Ageing Res Rev.

[3] de Joode SG, Kalmet PH, Fiddelers AA, Poeze M, Blokhuis TJ (2019). Long-term functional outcome after a low-energy hip fracture in elderly patients. J Orthop Traumatol.

[4] Chau DL, Walker V, Pai L, Cho LM (2008). Opiates and elderly: use and side effects. Clin Interv Aging.

[5] Pepe J, Ausman C, Tafti D, Madhani NB (2026). Ultrasound-guided fascia iliaca compartment block. StatPearls [Internet].

[6] Muse IO, Deiling B, Grinman L, Hadeed MM, Elkassabany N (2024). Peripheral nerve blocks for hip fractures. J Clin Med.

[7] Morrison RS, Dickman E, Hwang U (2016). Regional nerve blocks improve pain and functional outcomes in hip fracture: a randomized controlled trial. J Am Geriatr Soc.

[8] Page MJ, McKenzie JE, Bossuyt PM (2021). The PRISMA 2020 statement: an updated guideline for reporting systematic reviews. BMJ.

[9] Higgins JP, Altman DG, Gøtzsche PC (2011). The Cochrane Collaboration's tool for assessing risk of bias in randomised trials. BMJ.

[10] Wells G, Shea B, O'Connell D, Peterson J, Welch V, Losos M, Tugwell P (2026). The Newcastle-Ottawa Scale (NOS) for assessing the quality of nonrandomised studies in meta-analyses. http://www.ohri.ca/programs/clinical_epidemiology/oxford.asp.

[11] Moore TH, Higgins JP, Dwan K (2023). Ten tips for successful assessment of risk of bias in randomized trials using the RoB 2 tool: early lessons from Cochrane. Cochrane Evid Synth Methods.

[12] Sterne JA, Savović J, Page MJ (2019). RoB 2: a revised tool for assessing risk of bias in randomised trials. BMJ.

[13] Gualdi-Russo E, Zaccagni L (2026). The Newcastle-Ottawa Scale for assessing the quality of studies in systematic reviews. Publications.

[14] Ryan R, Hill S (2026). How to GRADE the quality of the evidence. Cochrane Consumers and Communication Group. https://colorectal.cochrane.org/sites/colorectal.cochrane.org/files/uploads/how_to_grade.pdf.

[15] Schünemann HJ, Higgins JP, Vist GE, Glasziou P, Akl EA, Skoetz N, Guyatt GH (2023). Completing 'Summary of findings' tables and grading the certainty of the evidence. Cochrane Handbook for Systematic Reviews.

[16] Bjelkarøy MT, Benth JŠ, Simonsen TB, Siddiqui TG, Cheng S, Kristoffersen ES, Lundqvist C (2024). Measuring pain intensity in older adults. Can the visual analogue scale and the numeric rating scale be used interchangeably?. Prog Neuropsychopharmacol Biol Psychiatry.

[17] Raiger LK, Gehlot RK, Bedi V, Betkeker SA (2019). Comparison of levobupivacaine and bupivacaine in fascia iliaca compartment block (FICB) for postoperative pain management in surgeries for fractures of neck of femur. Anaesth Pain Intensive Care.

[18] Xu L, Luo F, Lei E, Zhu X, Huang H, Li Q, Wan H (2020). The effect of the fascia iliaca compartment block combined with laryngeal mask general anesthesia on the internal fixation of senile femoral neck fracture. Int J Clin Exp Med.

[19] Sahithi Sahithi, Venkatraman T, Swetharamani R, Karthik C (2022). Krishnamoorthy: Evaluation of ultrasound-guided pre-emptive fascia iliaca compartment block for postoperative analgesia in femur and hip fracture surgeries: a randomised controlled trial. J Clin Diagn Res.

[20] Fahmy NM, Fouad G, Al Taher WA, Shabana TS, Ali SH (2026). Fascia iliaca block vs. multimodal analgesia for pain control following hip hemiarthroplasty. Anaesth Pain Intensive Care.

[21] Chen L, Liu S, Cao Y, Yan L, Shen Y (2023). Effect of perioperative ultrasound guided fascia iliaca compartment block in elderly adults with hip fractures undergoing arthroplasty in spinal anesthesia-a randomized controlled trial. BMC Geriatr.

[22] Nie H, Yang YX, Wang Y, Liu Y, Zhao B, Luan B (2015). Effects of continuous fascia iliaca compartment blocks for postoperative analgesia in patients with hip fracture. Pain Res Manag.

[23] Hao J, Dong B, Zhang J, Luo Z (2019). Pre-emptive analgesia with continuous fascia iliaca compartment block reduces postoperative delirium in elderly patients with hip fracture. A randomized controlled trial. Saudi Med J.

[24] Garlich JM, Pujari A, Moak Z (2020). Pain management with early regional anesthesia in geriatric hip fracture patients. J Am Geriatr Soc.

[25] Wang YL, Liu YQ, Ni H (2021). Ultrasound-guided, direct suprainguinal injection for fascia iliaca block for total hip arthroplasty: a retrospective study. World J Clin Cases.

[26] Salottolo K, Meinig R, Fine L (2022). A multi-institutional prospective observational study to evaluate fascia iliaca compartment block (FICB) for preventing delirium in adults with hip fracture. Trauma Surg Acute Care Open.

[27] Hao C, Li C, Cao R (2022). Effects of perioperative fascia iliaca compartment block on postoperative pain and hip function in elderly patients with hip fracture. Geriatr Orthop Surg Rehabil.

[28] Bali C, Ozmete O (2023). Supra-inguinal fascia iliaca block in older-old patients for hip fractures: a retrospective study. Braz J Anesthesiol.

[29] Dai X, Xing D, Luo J, Yang Y, Zhai J, Tang T, Yang W (2024). Fascia iliaca compartment block mitigates the fluctuations in heart rate variability and reduces pain with opioid consumption in elderly individuals with hip fractures: a randomized controlled trial. Heliyon.

[30] Caliskan B, Daglar S, Sen O (2025). Comparative efficacy of pericapsular nerve group and suprainguinal fascia iliaca blocks in elderly patients undergoing surgery for subtrochanteric femur fractures: a double-blind randomized controlled trial. Med Bull Haseki.

[31] Ma Y, Wu J, Xue J, Lan F, Wang T (2018). Ultrasound-guided continuous fascia iliaca compartment block for pre-operative pain control in very elderly patients with hip fracture: a randomized controlled trial. Exp Ther Med.

[32] Bang S, Chung J, Jeong J, Bak H, Kim D (2016). Efficacy of ultrasound-guided fascia iliaca compartment block after hip hemiarthroplasty: a prospective, randomized trial. Medicine (Baltimore).

[33] McGuinness LA, Higgins JP (2021). Risk-of-bias VISualization (robvis): an R package and Shiny web app for visualizing risk-of-bias assessments. Res Synth Methods.

[34] Hartling L, Milne A, Hamm MP, Vandermeer B, Ansari M, Tsertsvadze A, Dryden DM (2013). Testing the Newcastle Ottawa Scale showed low reliability between individual reviewers. J Clin Epidemiol.

[35] Centers for Disease Control and Prevention (2024). Integrating randomized and nonrandomized studies in evidence synthesis. ACIP GRADE Handbook for Developing Evidence-Based Recommendations.

[36] Mitchell AJ, Shukla D, Ajumal HA, Stubbs B, Tahir TA (2014). The Mini-Mental State Examination as a diagnostic and screening test for delirium: systematic review and meta-analysis. Gen Hosp Psychiatry.

[37] Khor HM, Ong HC, Tan BK (2019). Assessment of delirium using the confusion assessment method in older adult inpatients in Malaysia. Geriatrics (Basel).

[38] Wong EK, Lee JY, Surendran AS (2018). Nursing perspectives on the confusion assessment method: a qualitative focus group study. Age Ageing.

[39] Sheehan KJ, Goubar A, Almilaji O (2021). Discharge after hip fracture surgery by mobilisation timing: secondary analysis of the UK National Hip Fracture Database. Age Ageing.

[40] Garg R, Singh S, Kumar A, Kumar G (2025). Comparative analysis of bupivacaine vs. ropivacaine in peripheral nerve blocks. Int J Acad Med Pharm.

[41] Turabi RY, Sheehan KJ, Guerra S, O'Connell MD, Wyatt D (2024). Barriers and facilitators to early mobilisation and weight-bearing as tolerated after hip fracture surgery among older adults in Saudi Arabia: a qualitative study. Age Ageing.

[42] Paunikar S, Chakole V (2024). Postoperative delirium and neurocognitive disorders: a comprehensive review of pathophysiology, risk factors, and management strategies. Cureus.

[43] Mosaffa F, Taheri M, Manafi Rasi A, Samadpour H, Memary E, Mirkheshti A (2022). Comparison of pericapsular nerve group (PENG) block with fascia iliaca compartment block (FICB) for pain control in hip fractures: a double-blind prospective randomized controlled clinical trial. Orthop Traumatol Surg Res.

